# Activation of cryptic metabolite production through gene disruption: Dimethyl furan-2,4-dicarboxylate produced by *Streptomyces sahachiroi*

**DOI:** 10.3762/bjoc.9.205

**Published:** 2013-08-29

**Authors:** Dinesh Simkhada, Huitu Zhang, Shogo Mori, Howard Williams, Coran M H Watanabe

**Affiliations:** 1 Texas A&M University, Department of Chemistry, College Station, TX 77843, USA

**Keywords:** cryptic metabolite, dimethyl furan-2,4-dicarboxylate, genetic knockout, natural products, non-ribosomal peptide synthetase module, *Streptomyces*

## Abstract

At least 65% of all small molecule drugs on the market today are natural products, however, re-isolation of previously identified and characterized compounds has become a serious impediment to the discovery of new bioactive natural products. Here, genetic knockout of an unusual non-ribosomal peptide synthetase (NRPS) C-PCP-C module, *aziA2*, is performed resulting in the accumulation of the secondary metabolite, dimethyl furan-2,4-dicarboxylate. The cryptic metabolite represents the first non-azinomycin related compound to be isolated and characterized from the soil bacterium, *S. sahachiroi*. The results from this study suggest that abolishing production of otherwise predominant natural products through genetic knockout may constitute a means to “activate” the production of novel secondary metabolites that would otherwise lay dormant within microbial genome sequences.

## Findings

It has become increasingly apparent that microbial diversity in nature far exceeds that reflected in laboratory strain collections. The advent of microbial genomics and the availability of published genome sequences suggest, that as much as 90% of the chemical potential of these organisms remain “silent” within the genome [1–2]. While the biosynthetic machinery appears functional, the genetic loci are frequently (but not always) unexpressed. The activation of such pathways has garnered interest from the scientific community. Quorum sensing refers to the process by which microorganisms communicate (within a single bacterial species or between diverse species) and alter their gene expression through production of certain signaling molecules [3]. In this regard, the culturing of microbial communities or the co-culture of two specific microbial organisms has been successfully implemented to “activate” secondary metabolite production [4–5]. In some instances, shot-gun cloning and expression of soil samples and bacterial symbiont genomes have led to the discovery of a representative set of natural products and enzymes, albeit typically of small biosynthetic pathways (less than 10 kb) [6–7]. Direct manipulation, “induced” biosynthetic activation, of a sequenced cluster has also met with some success. The *Aspergillus nidulans* genome was mined for cryptic orphan gene clusters from which a single, unexpressed PKS-NRPS hybrid was identified. The expression of the gene cluster was activated by the ectopic expression of a specific regulatory gene, *apdR*, resulting in the production of the aspyridones [8]. Sometimes, a cryptic pathway is fully expressed and has simply gone unnoticed. In this regard, a genome-isotopic approach has proven useful where stable isotope-labeled putative precursors of the orphan pathway are fed to the organism and used to screen extracts of the fermentation broth, to identify metabolites containing the labeled precursors [9–10].

**Figure 1:**
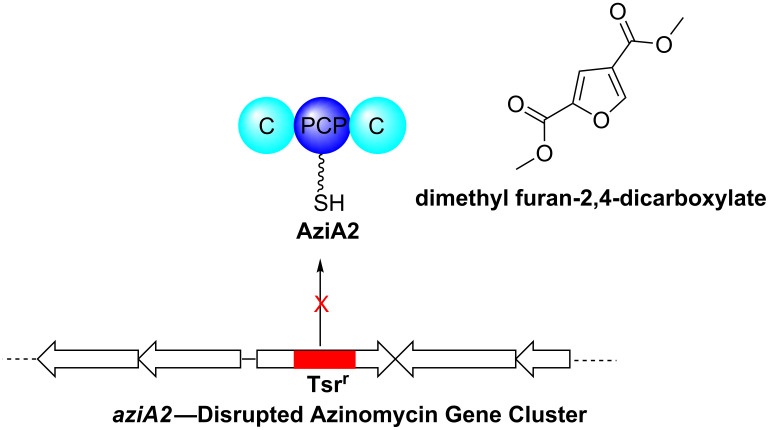
Schematic diagram illustrating the phenotypic effects arising through deletion of the *aziA2* gene.

Here, we have carried out a functional knockout of *aziA2* within the azinomycin gene cluster of *Streptomyces sahachiroi* [11–12]. The gene encodes a NRPS (non-ribosomal peptide synthetase, an enzyme involved in the biosynthesis of various peptide containing secondary metabolites) module of unusual domain architecture consisting of condensation, peptidyl carrier protein, and condensation domains (C-PCP-C), respectively. Gene inactivation led to the abolishment of azinomycin production, confirming the involvement of *aziA2* in the biosynthesis of the antitumor agent [11,13] and also led to overproduction of a compound, dimethyl furan-2,4-dicarboxylate. The metabolite represents one of many cryptic pathway metabolites from the *S. sahachiroi* genome and is the first non-azinomycin related compound to be isolated from the *S. sahachiroi* strain (Figure 1). Previously isolated metabolites consisted of azinomycin intermediates lacking the 1-azabicyclo[3.1.0] ring system, including naphthoate and naphthoate epoxide derivatives [11].

**Figure 2:**
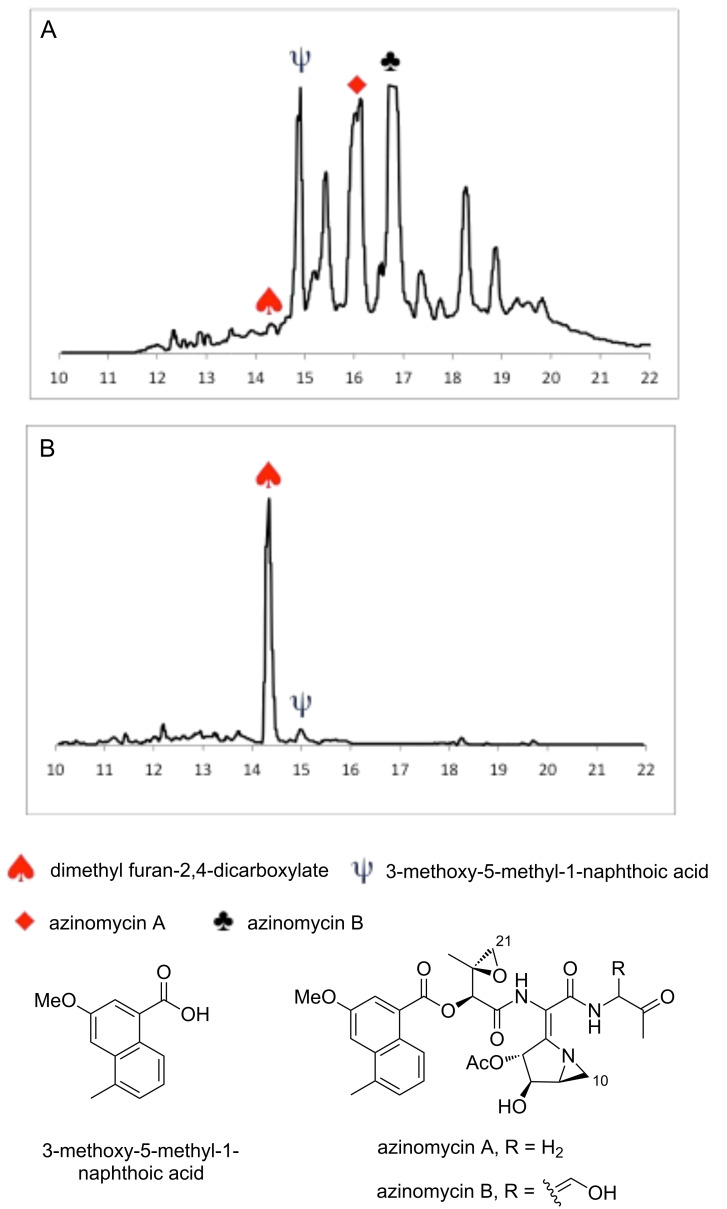
HPLC profiles of (A) *S. sahachiroi* wild-type and (B) *ΔaziA2* crude organic extracts.

The *aziA2* disruption construct (pKC-AziA2) was generated by replacing the mid-portion (1500 bp) of the gene with a thiostrepton resistance marker within the vector pKC1139. The plasmid was introduced into *S. sahachiroi* by conjugal transformation. Single crossover mutants were initially selected by culturing the strain on both apramycin and thiostrepton MS-agar plates. The double crossover mutants were subsequently generated from the single crossover mutants by growing the colonies on MS-agar plates in the presence of only thiostrepton antibiotic, resulting in the occurrence of double crossover events. The *S. sahachiroi*/Δ*aziA2* disruptants were confirmed by PCR and Southern hybridization of thiostrepton resistant and apramycin sensitive colonies. Direct comparison of HPLC profiles of the wild-type and *ΔaziA2* crude organic extracts were striking (Figure 2). The *ΔaziA2* mutant gave a single major peak. 3-Methoxy-5-methyl-1-naphthoic acid was detected in trace amounts (suggesting that the metabolite is released in solution from the PKS and not directly transferred to that of a subsequent NRPS module) and complete abolishment of the azinomycins was observed. The production of the naphthoic acid derivative and lack of azinomycin production confirms the involvement of *aziA2* (C-PCP-C NRPS module) in azinomycin biosynthesis; given the unique domain architecture of this module, this is a significant finding as non-functional NRPS modules have been found imbedded in certain gene clusters [14].

Structural characterization of the major metabolite was carried out using LC–MS analysis and 1D and 2D cryoprobe NMR spectroscopy (data provided in Supporting Information File 1). The molecular formula was established as C_8_H_8_O_5_ based upon a molecular ion peak observed by LC–APCIMS at *m*/*z* 185.0453 [M + H]^+^ and GC–MS. The GC–MS fragmentation of the compound matched that provided in the Ultra GC–DSQ (ThermoElectron, Waltham, MA) GC–MS database. The structure of the compound was determined by NMR analysis in CDCl_3_ (Table 1). The presence of two O-CH_3_ groups at C-2’ (δ_c_ 51.99, δ_H_ 3.94) and C-2” (δ_c_ 52.31, δ_H_ 3.88) and double bonds in the aromatic ring at C-3 (δ_c_ 117.07, δ_H_ 7.49, d, *J* = 0.811) and C5 (δ_c_ 150.14 δ_H_ 8.14, d, *J* = 0.845) were observed by ^1^H NMR, DEPT, HMQC and HMBC. C-1” (δ_c_ 158.53), C-2 (δ_c_ 145.44), C-5 (δ_c_ 150.14) and C-1’ (δ_c_ 162.34) signals were detected by ^13^C NMR (Table 1). The data identified the compound as dimethyl furan-2,4-dicarboxylate, which has been previously observed in microbial head-space or vapor phase extracts and its structure determined through chemical synthesis [15]. The biosynthetic origin of dimethyl furan-2,4-dicarboxylate could be polyketide derived where the furan ring system could be envisioned to form through nucleophilic attack of an epoxide as in the case with monensin and other similar natural products [16]. Alternatively, the biosynthesis might involve the dimerization of pyruvate [17–19]. Examination of a draft genome sequence of the *S. sahachiroi* strain has implicated a pyruvate aldolase and methyltransferase containing gene cluster as well as potential polyketide gene clusters containing methyltransferase genes, which will be evaluated in due course through genetic knockout experiments of the *ΔaziA2* mutant strain.

**Table 1:** ^13^C NMR (δ_C_) and ^1^H NMR (δ_H_, *J* in Hz) 500 MHz spectroscopic data in CDCl_3_.

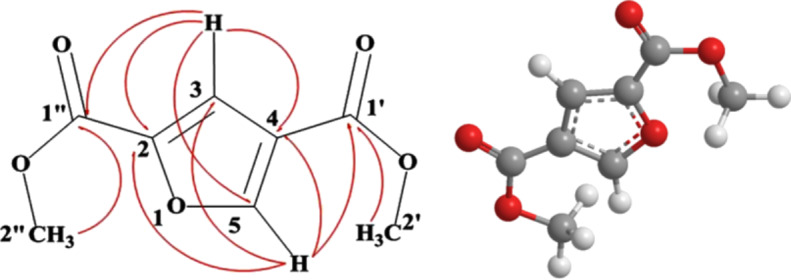			

Position	δ_C_	δ_H_ (*J* in Hz)	HMBC (H→C)

1			
2	145.44, CO		
3	117.07, CH	7.49, (0.811) d	2,4,5,1”
4	120.96, C		
5	150.14, CH	8.14, (0.845) d	2,3,4,1’
1’	162.34, C		
1”	158.53, C		
2’	51.99, CH_3_	3.94, s	1”
2”	52.32, CH_3_	3.88, s	1’

Disruption of the *aziA2* gene gave overproduction of the cryptic metabolite dimethyl furan-2,4-dicarboxylate and led to complete abolishment of azinomycin production. It is the first non-azinomycin related metabolite to be reported from the *S. sahachiroi* strain, a microorganism that has been mined for its natural product constituents since 1954 [20]. These results demonstrate that while some disruption strains will certainly be more easily characterized than others, the act of simply inactivating genes from highly expressed or represented biosynthetic gene clusters can be a useful strategy toward revealing or unmasking novel natural products or cryptic metabolites for subsequent isolation and characterization. Secondary metabolite production by microorganisms is often viewed as a line of defense against competing microbes for nutrients. Natural products like azinomycin, a DNA crosslinking agent, can offer a means of protection to the producing organism. It is tempting to speculate that when biosynthetic pathways of these metabolites are inactivated, the microorganism begins to adapt and evolve in an effort to develop new self defense mechanisms. Such adaptations (which may or may not be immediately useful to the host) can lead to the production of new metabolites such as dimethyl furan-2,4-dicarboxylate.

## Experimental

### DNA manipulation and methods

Conventional recombinant DNA techniques were utilized for plasmid and genomic DNA isolation, restriction enzyme digestion, agarose gel electrophoresis, and DNA ligations [21]. DNA sequencing was performed using ABI BigDye chemistry at the Gene Technologies Lab, Texas A&M University. The computer-based analysis and comparisons of nucleotide and protein sequences were performed with BLAST, FASTA, CLUSTALW and GENEDOC programs. See Supporting Information File 1 for details on bacterial strains, plasmids, PCR primer sequences, and bacterial culture conditions.

### Construction of disruption plasmid pKCAziA2

Disruption of *aziA2* was performed using a homologous recombination approach with pKC1139. PCR with primer pairs aziA2UF/aziA2UR and aziA2DF/aziA2DR was utilized to generate a 930 bp upstream fragment and 923 bp downstream fragment relative to *aziA2*. Amplification was carried out as follows: initial denaturation at 98 °C for 30 s; 30 total cycles of denaturation for 10 s; annealing at 66.4 °C for aziA2UF/aziA2UR and 62 °C for aziA2DF/aziA2DR for 40 s; polymerization at 72 °C for 60 s and finally gap filing at 72 °C for 10 min. The amplified DNA fragment of azia2-U was digested with *Hind*III/*Xba*I (azia2-U) and *Xba*I/*Eco*RI (azia2-D) and cloned into the corresponding sites of pKC1139, resulting in pKCA2UD. pKCA2UD was digested with *Xba*I and ligated with the thiostrepton-resistance gene (1056 bp), which was amplified with TsrF/TsrR primers using the same amplification condition as detailed for aziA2DF/aziA2DR, to give the final disruption recombinant plasmid pKC-AziA2, where *aziA2* is replaced by tsr^r^ in-frame.

### Conjugal transformation with plasmid pKCAziA2

pKC-AziA2 was transformed into *E. coli* S-17, a donor host and subsequently transferred into wild type, *S. sahachiroi* by conjugal transformation, as described by Kieser et al. (with some modification) [22] for deletion with a replicative plasmid as mediated by homologous recombination. From an overnight 5 mL culture of *E. coli* donor strain, 100 µL was transferred into 100 mL fresh LB medium and cultured for 4 h. The cells at OD600 0.6 were harvested, washed twice and solubilized in 500 µL of 2xYT medium. Spores of *S. sahachiroi* were prepared in MS agar plates and collected after one week. Spores were washed, heat shocked at 70 °C for 10 min and incubated at 37 °C for 4 h. Following incubation, they were washed and resuspended in 500 µL of 2xYT medium. These recipient cells were mixed with an equal volume of *E. coli* donor cells and 200 µL of the mixture were plated on MS-agar plates. The plates were incubated at 30 °C for 18 h, and overlayed with 1 mL water containing nalidixic acid (500 µg), to inhibit the growth of *E. coli*, and apramycin (25 µg) to select *S. sahachiroi* exconjugants. Incubation at 30 °C was continued for 6–8 days to allow growth of the exconjugants. Apramycin exconjugants were screened for several generations until the exconjugants were found to be apramycin sensitive and thiostrepton resistant to detect the double crossover allelic exchange. For further confirmation of deletion of *aziA2* from *S. sahachiroi*, genomic DNA was isolated from both *S. sahachiroi* wild-type and *aziA2* knockout strains (*S. sahachiroi*/*ΔaziA2*) and PCR performed. The AziA2UF/TsrR and TsrF/AziA2DR primer set was utilized: initial denaturation at 98 °C for 30 s; 30 total cycles of denaturation for 10 s; annealing at 64.5 °C for 40 s; polymerization at 72 °C for 60 s and finally gap filing at 72 °C for 10 min. Southern hybridization was carried out following standard procedures [21] with a radioactive ^32^P-ATP labeled probe. The probe was generated through PCR amplification using the *aziA2* probe-F/aziA2 Probe R set of primers and following the same PCR condition as detailed above.

### Fermentation and analysis of secondary metabolites

Fermentation was carried out in 10 L following established procedures [23]. After 72 h of incubation, the culture broth was centrifuged and the supernatant extracted with dichloromethane (2× the volume of the medium). The organics were concentrated in vacuo and analyzed by reversed-phase HPLC. The column, Phenomenex, Columbus 5µ C8 100A (250 × 3.20 mm 5 µ) was equilibrated with 10% of A (75% MeOH/25% isopropanol) and 90% of B (water) and run with the following program: Time 0 min A-10%; 1 min A-10%; 5 min A-35%; 14 min A-95%; 15 min A-95%; 20 min A-10%; 25 min A-10%; 60 min A-100%. This was carried out at a flow rate of 0.75 mL/min in 254 nm UV. The same program was used for LC–APCI analysis. For GC–MS analysis, the sample was diluted 1:10 in DCM and was performed on an Ultra GC/DSQ (ThermoElectron, Waltham, MA). A Rxi-5ms gas chromatographic column was utilized with dimensions of 60 m length, 0.25 mm i.d., and 0.25 μm film thickness (Restek; Bellefonte, PA). Helium was implemented as a carrier gas at a constant flow rate of 1.5 mL/min. The transfer line and ion source were held at 250 °C. The column temperature was maintained at 50 °C for 5 min then raised to 320 °C for 20 °C/min. Mass spectra were acquired in full scan mode in the range of 30–500 *m*/*z*.

### Cryoprobe NMR analysis

NMR spectra were acquired on a Bruker Avance III 500 MHz instrument at 25 °C, equipped with a 5 mm H–C–N cryoprobe. The sample was dissolved in 125 μL CDCl_3_ and contained in a 3 mm wilmad 335 pp NMR tube. Proton, carbon, Dept 90, Dept 135, HMQC and HMBC spectra were recorded. A 30° observe pulse was used to acquire the ^1^H NMR spectrum, while a 45^o^ pulse was used to obtain the ^13^C NMR spectrum.

## Supporting Information

File 1 Details on genetic knockout, Southern blot analysis, LC–MS analysis and NMR data.
