# Supplementary material for: Activation of cryptic metabolite production through gene disruption: Dimethyl furan-2,4-dicarboxylate produced by Streptomyces sahachiroi
Source: Beilstein J Org Chem. 2013 Aug 29;9:1768–73. doi: 10.3762/bjoc.9.205 (PMC3778384; doi:10.3762/bjoc.9.205)
Supplement: File 1 — Details on genetic knockout, Southern blot analysis, LC–MS analysis and NMR data. [file Beilstein_J_Org_Chem-09-1768-s001.pdf]

Supporting Information

for

Activation of cryptic metabolite production through gene disruption: Dimethyl furan-2,4-dicarboxylate produced by *Streptomyces sahachiroi*

Dinesh Simkhada, Huitu Zhang, Shogo Mori, Howard Williams, Coran M. H. Watanabe*

Address: Texas A&M University, Department of Chemistry, College Station, TX 77843, USA

Email: Coran M. H. Watanabe – watanabe@chem.tamu.edu

* Corresponding author

Details on genetic knockout, Southern blot analysis, LC–MS analysis and NMR data

Contents

Figure S1: Strategy for genetic knockout of *aziA2*……………………………………………..S3

Figure S2: Confirmation of disruptants: Southern blot……….…………………………......S4

Figure S3: NMR profile analysis of dimethyl furan-2,4-dicarboxylate (in order: ^{1}H NMR, ^{13}C NMR, Dept 90, Dept 135, HMBC and HMQC)………………………………………..S5–S10

Figure S4: LC–APCIMS profile of dimethyl furan-2,4-dicarboxylate……….…..………...……S11

Figure S5: LC–APCIMS/MS profile of dimethyl furan-2,4-dicarboxylate……………………...S11

Figure S6: GC–MS profile of dimethyl furan-2,4-dicarboxylate……………………………...S12

Supplemental experimental procedures…………………………………………...………S13

Table S1: List of strains and plasmids used in this study……………………………..……..S13

Table S2: List of primers used in this study…………………………………………………...S14

References…………………………………………………………..…….......................S15

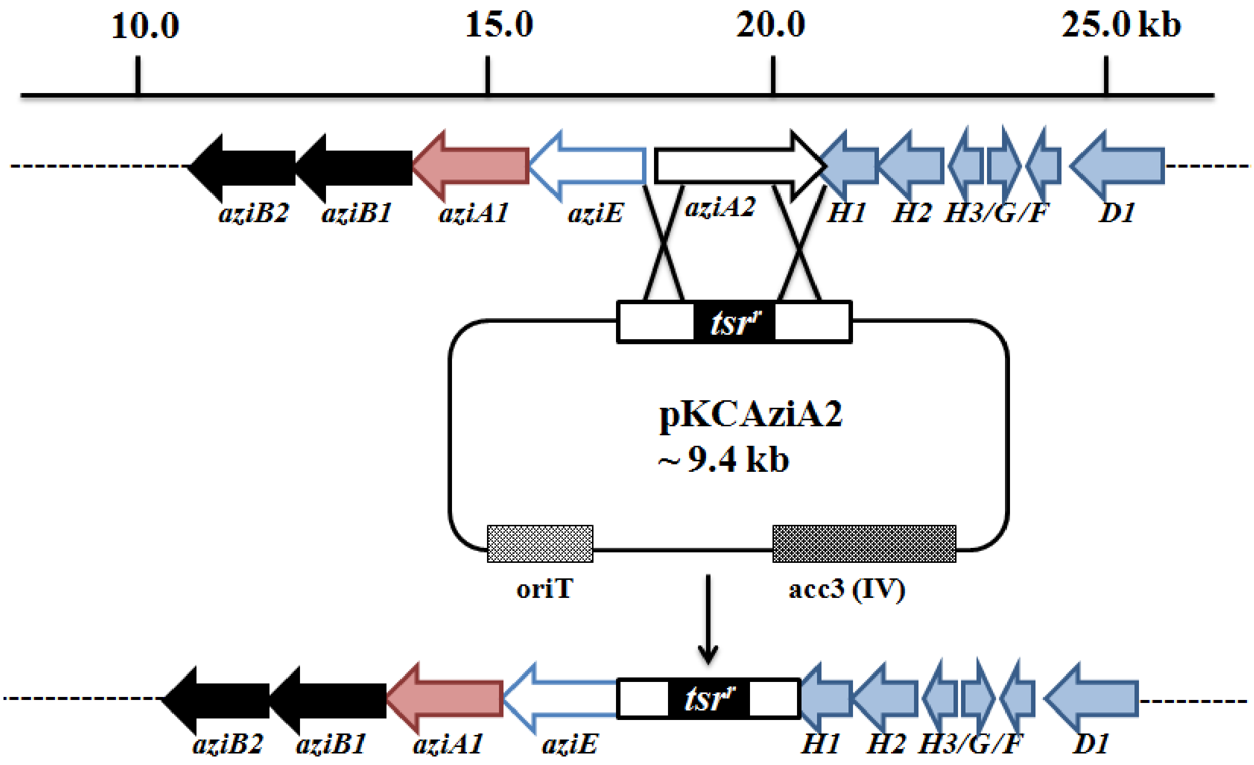

Figure S1: Strategy for genetic knockout of *aziA2.*

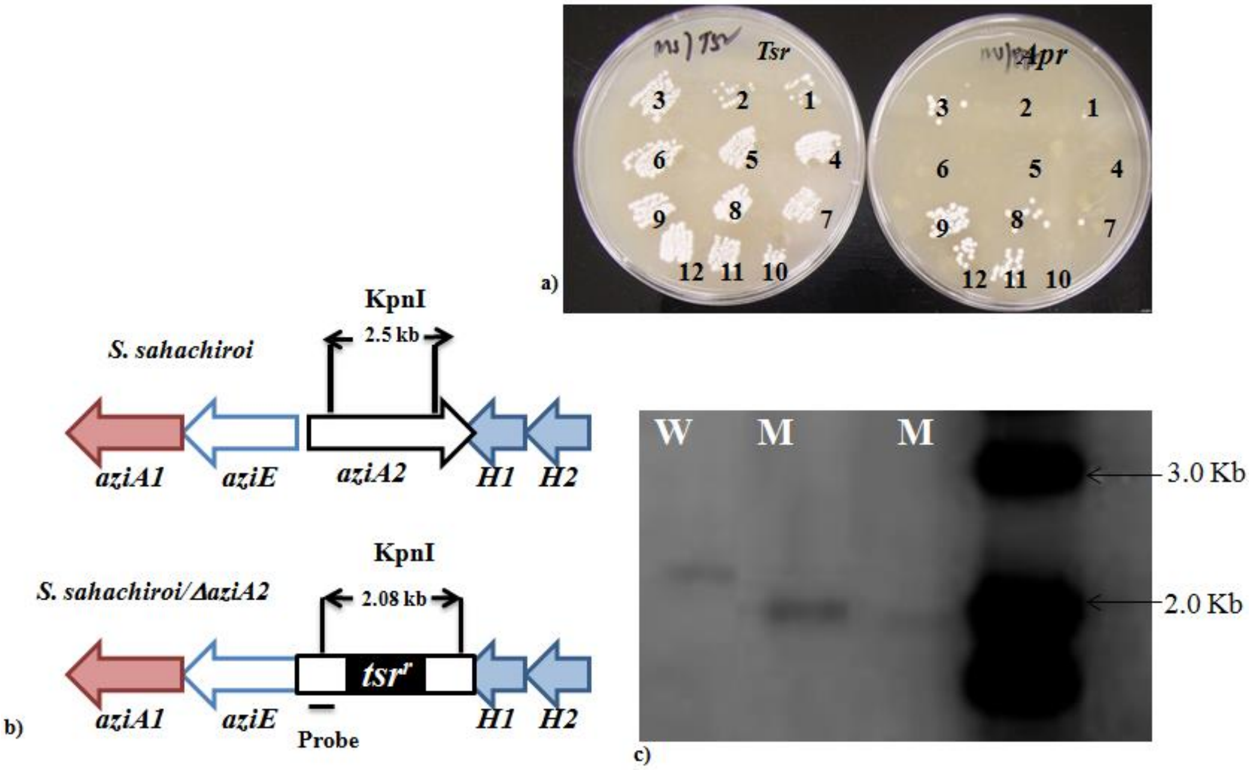

Figure S2: Confirmation of *aziA2* knockout: a) Screening for positive colonies, b) Strategy for Southern hybridization, c) Southern blot; W: wild type, M: mutants.

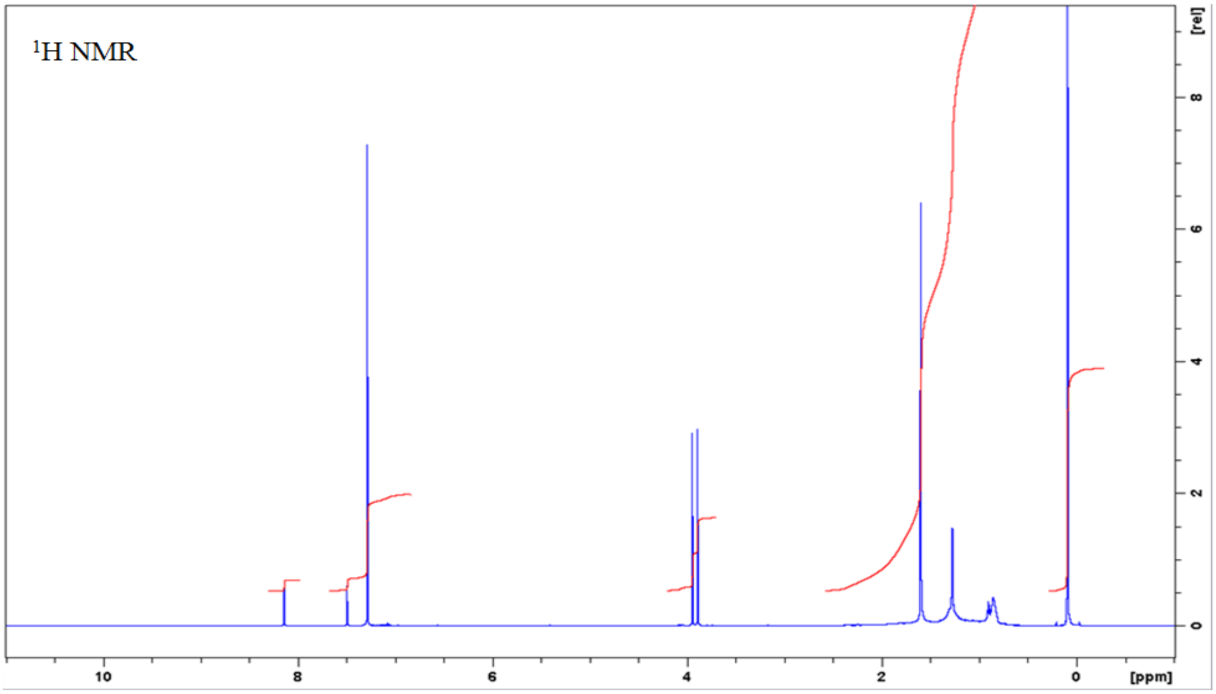

¹H NMR
[rel]
8
6
4
2
0
10
8
6
4
2
0
[ppm]

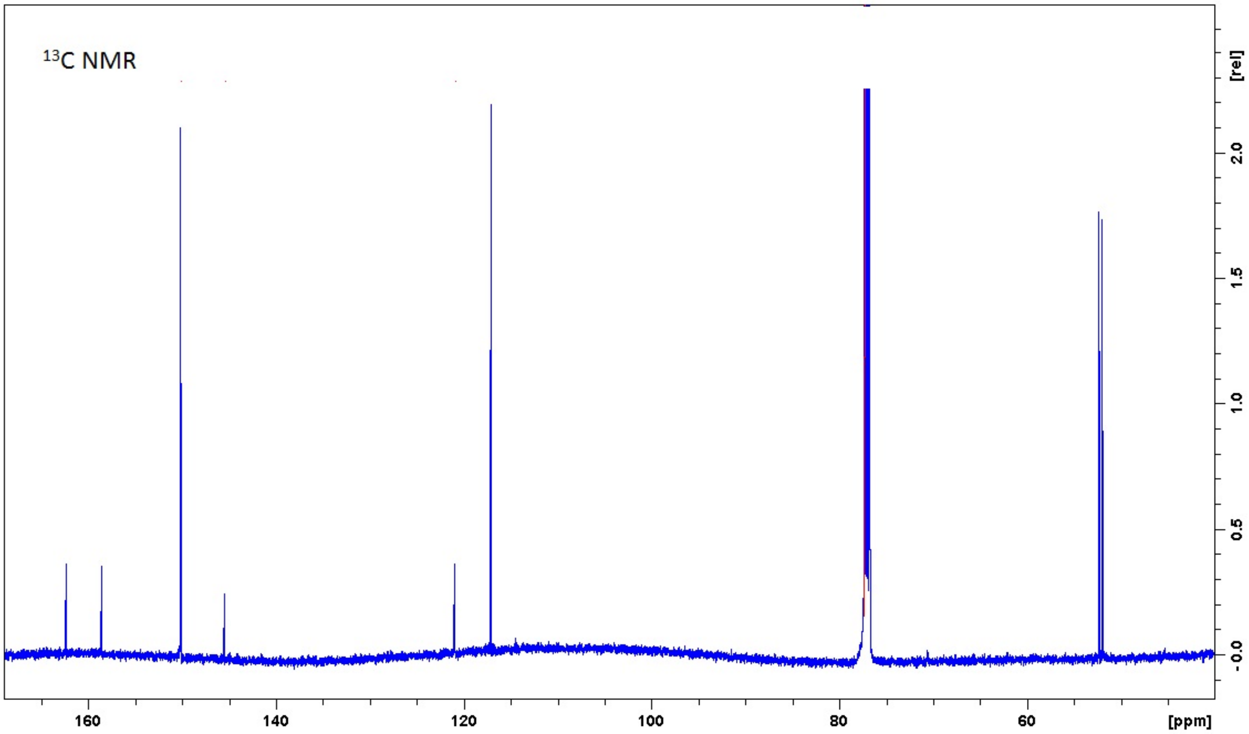

¹³C NMR
[rel]
2.0
1.5
1.0
0.5
-0.0
160
140
120
100
80
60
[ppm]

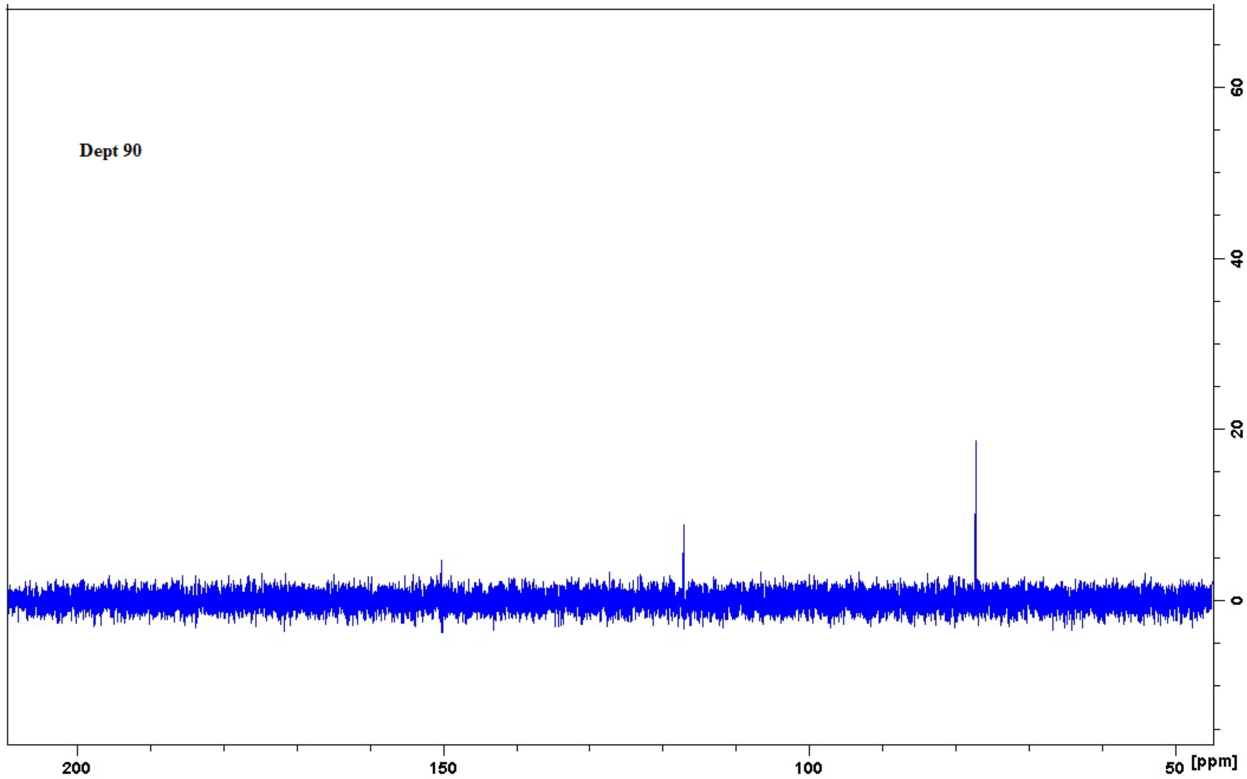
Dept 90
200
150
100
50
[ppm]
60
40
20
0

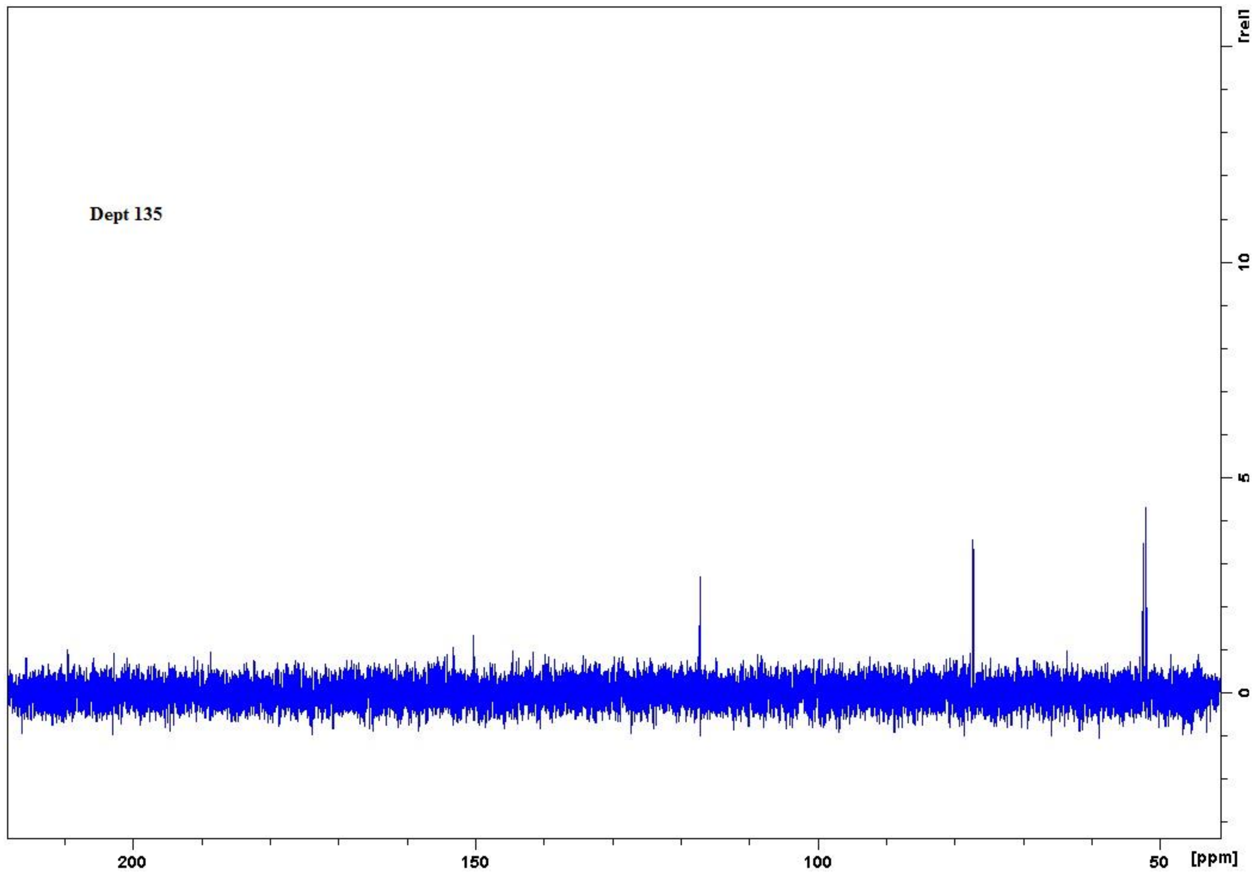
Dept 135
200
150
100
50
[ppm]
[rel]
10
5
0

HMBC (1)

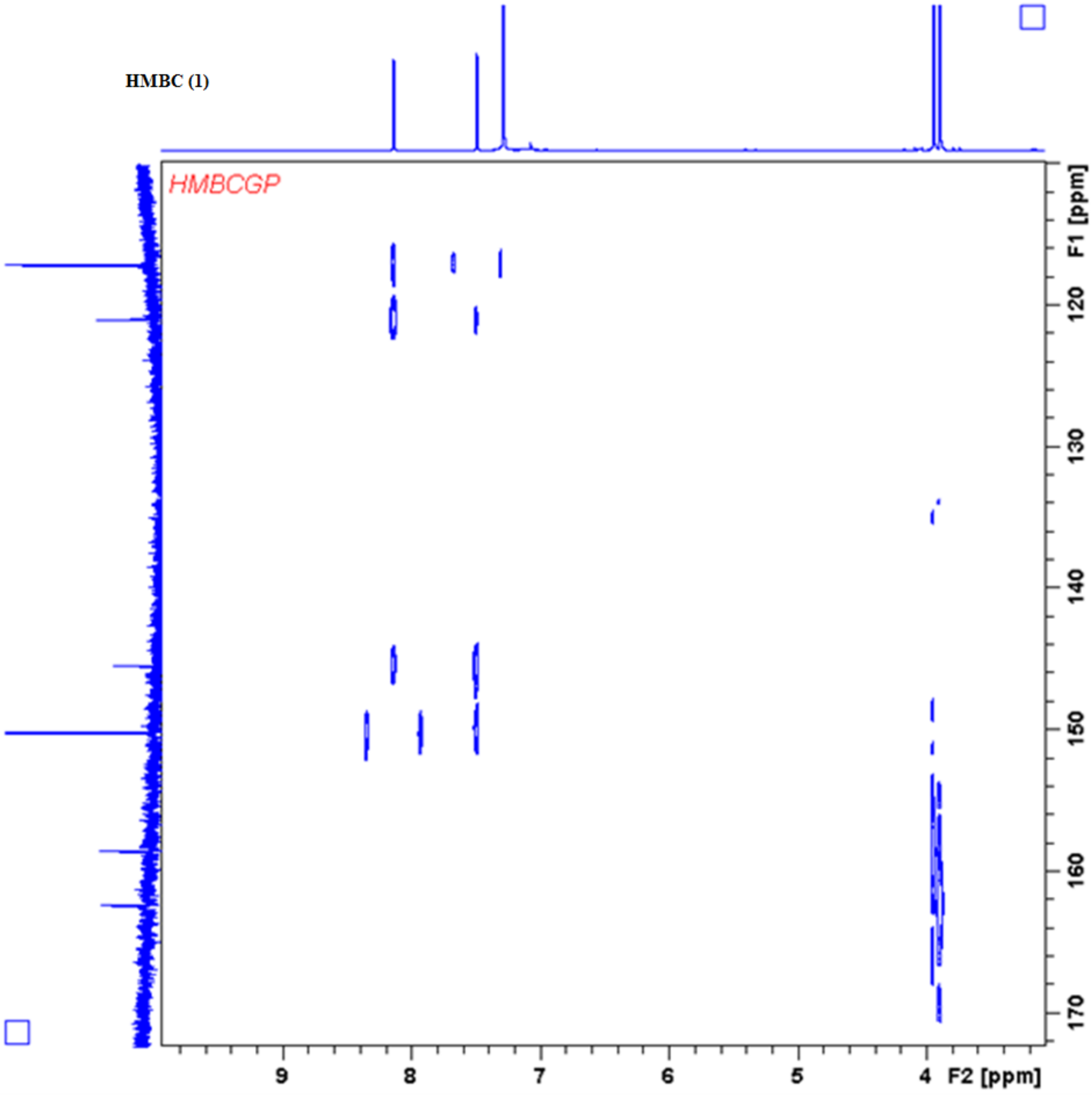

HMBC (2)

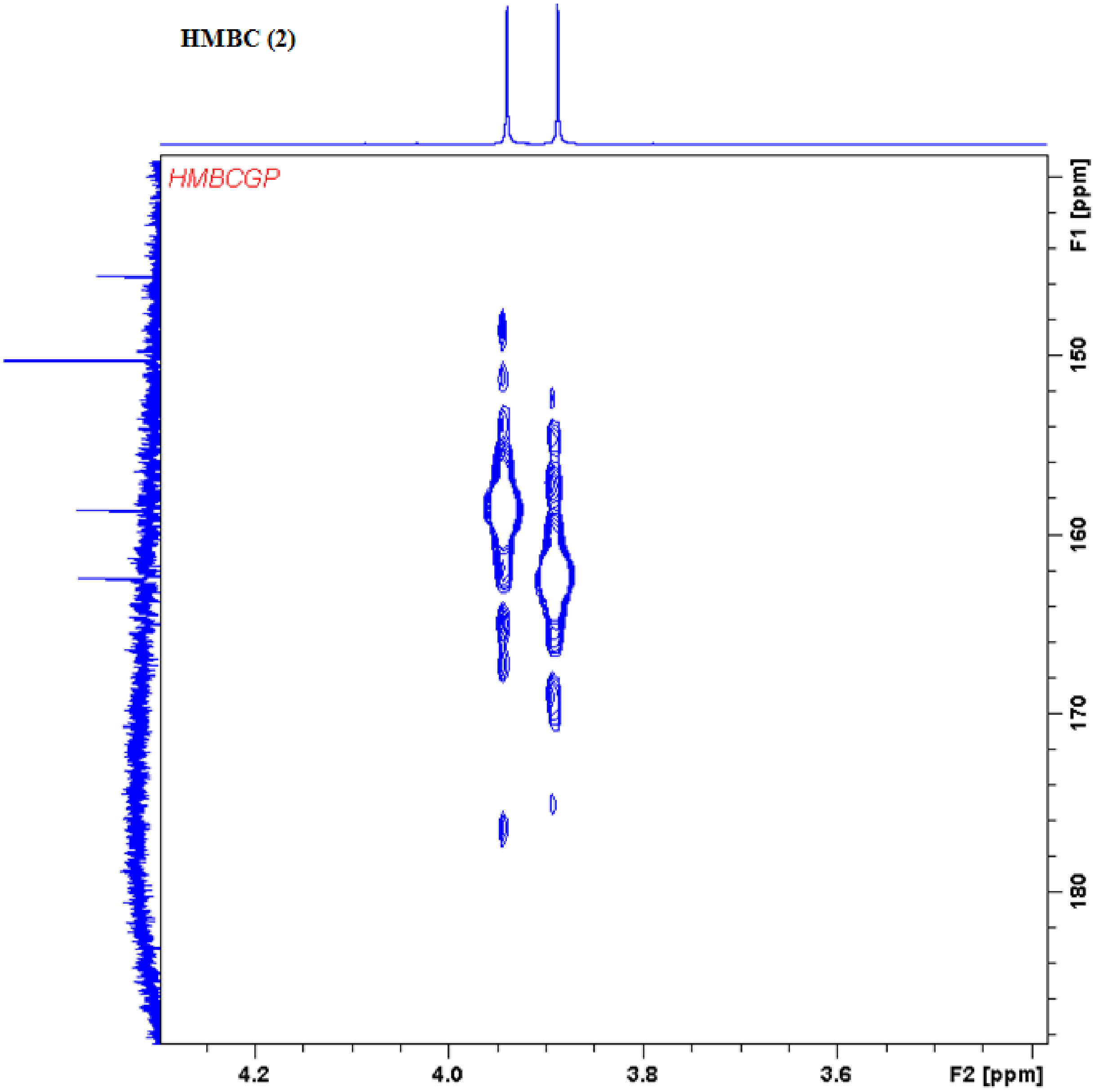

HMBC (3)

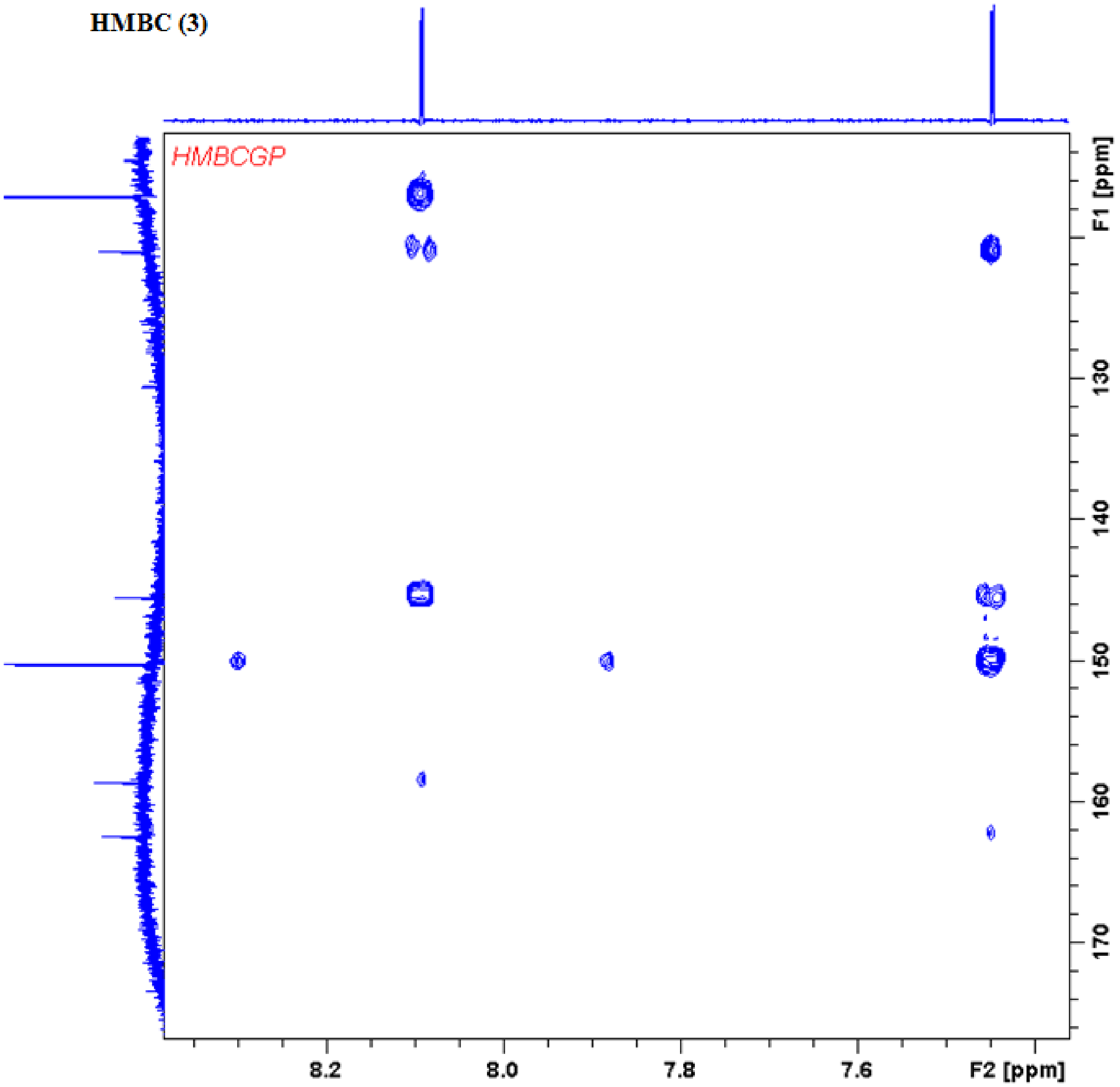

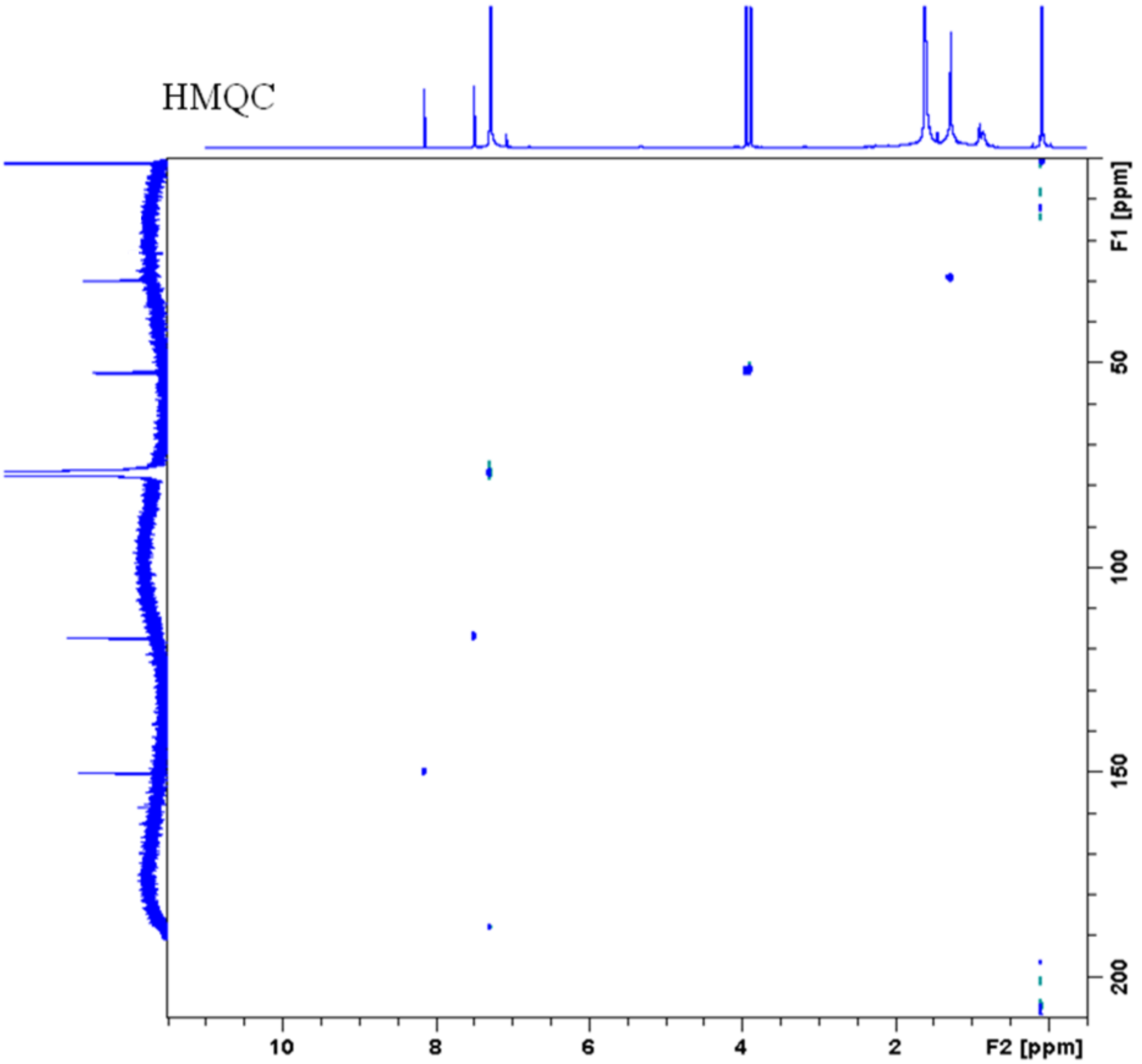

Figure S3: NMR Profile for dimethyl furan-2,4-dicarboxylate (in order: ^{1}H NMR, ^{13}C NMR, Dept 90, Dept 135, HMBC (1,2 and 3) and HMQC).

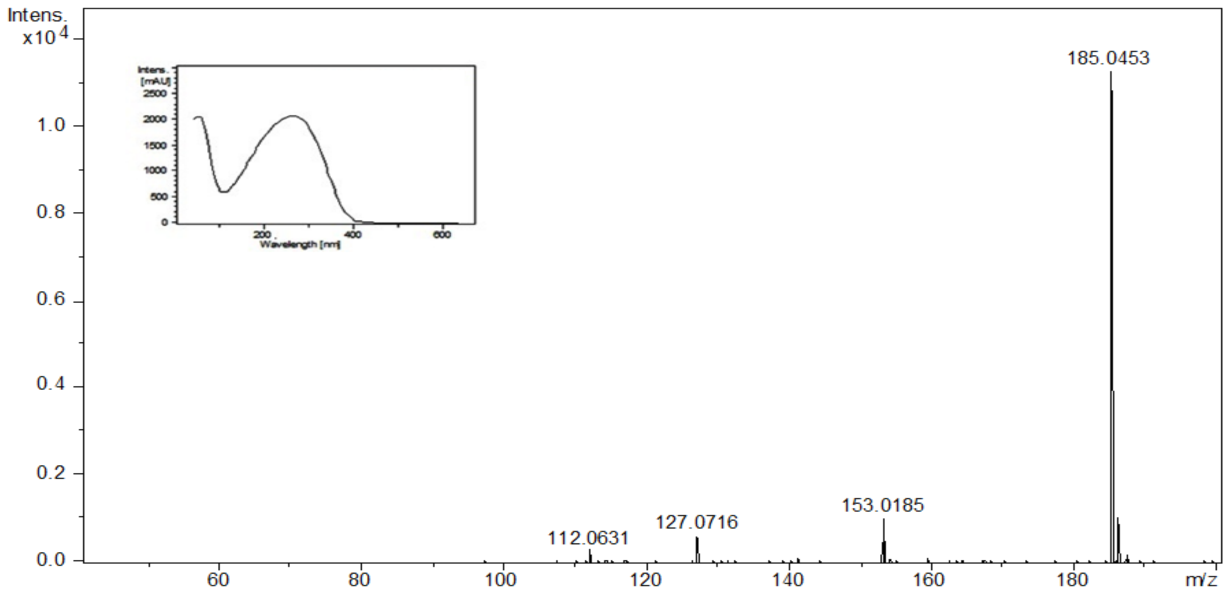

Figure S4: LC–APCIMS profile of dimethyl furan-2,4-dicarboxylate.

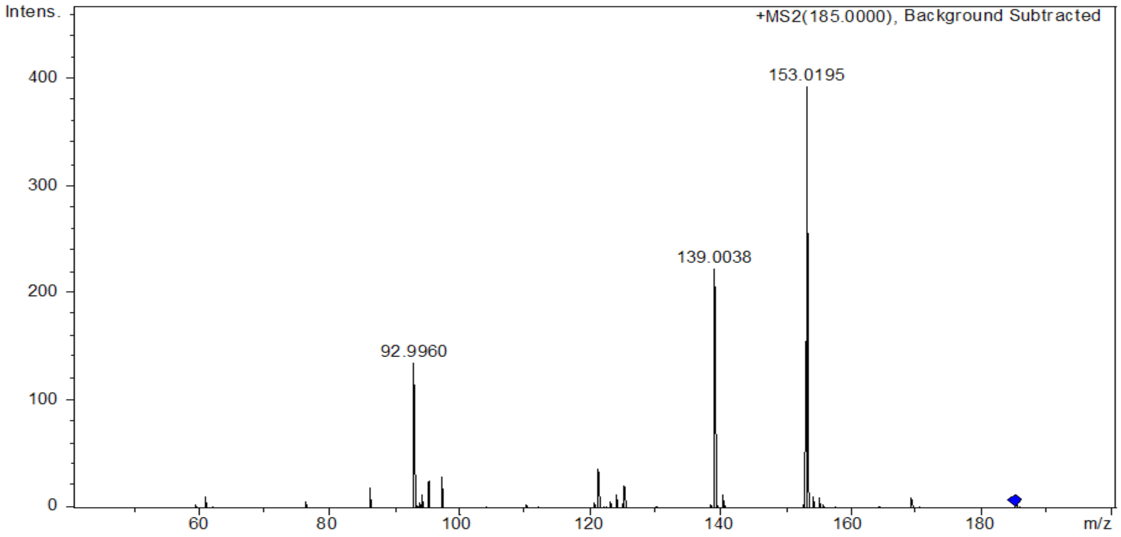

Figure S5: LC–APCIMS/MS profile of dimethyl furan-2,4-dicarboxylate.

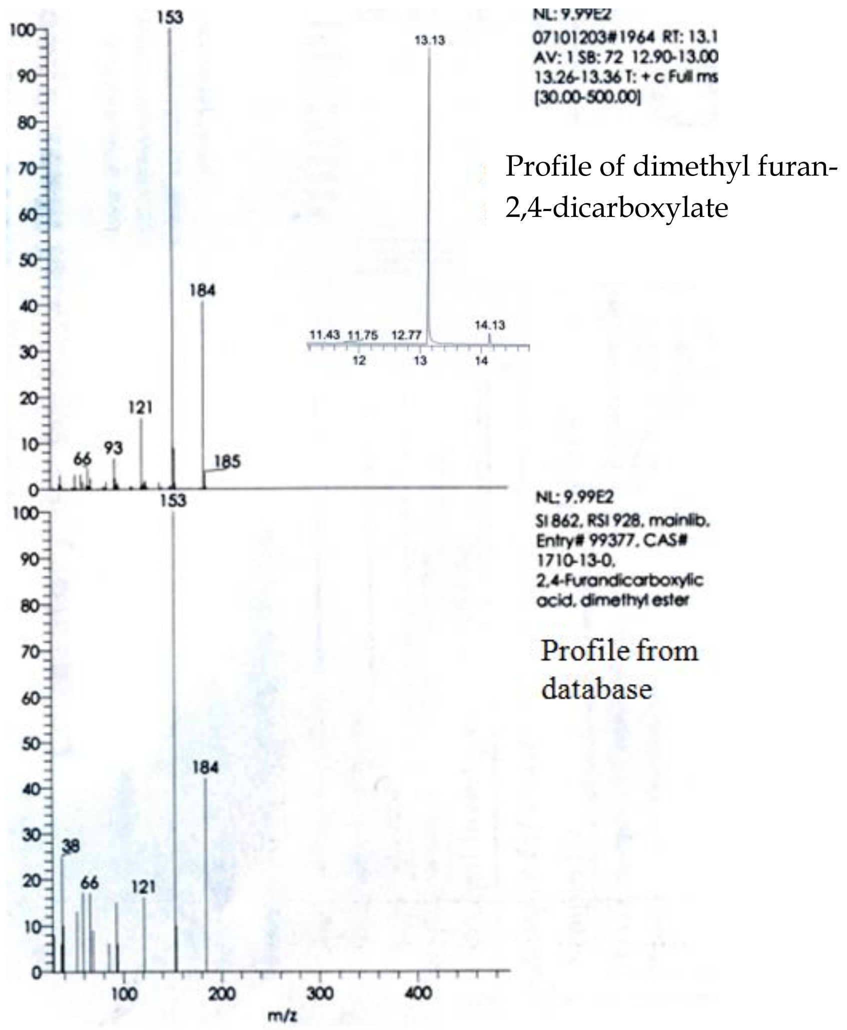

Figure S6: GC–MS profile of dimethyl furan-2,4-dicarboxylate.

Supplemental experimental procedures

Bacterial strains, plasmids and culture conditions

Escherichia coli (*E. coli*) strains (XL1 Blue and DH10B) were cultured in Luria Bertani (LB) medium at 37 °C. *E. coli* XL1 Blue was routinely used as a host for the recombinant plasmids. *E. coli* S17-1 was used as a donor strain for conjugative transfer of mobilizable plasmids into *Streptomyces sahachiroi*. The parental strain *S. sahachiroi* was cultured at 28 °C in R2YE medium [1] for 2–3 days for the preparation of genomic DNA. pGEM-T Easy (Promega, USA) was used as a cloning vector and pKC1139 was used as disruption vector. All of the bacterial strains and a list of vectors and recombinant plasmids including their relevant sources are provided in Table S1.

Table S1: List of strains and plasmids used in this study.

Strains/Plasmids	Relevant characteristics	References
**Strains**		
E. coli XL1 Blue	*recA1 endA1 gyrA96 thi-1 hsdR17 supE44 relA1 lac* [F' *proAB lacI ^{q} Z DM15* Tn*10* (Tet^r)]	Stratagene La Jolla, CA, USA
E. coli S17-1	*recA1 pro thi*; has the *tra* genes from plasmid RP4 integrated in the chromosome	Simon *et. al*., 1983 [2]
E. coli S17-1/pKC-AziA2	*recA1 pro thi*; has the *tra* genes from plasmid RP4 integrated in the chromosome with *aziA2* disruption plasmid pKC-AziA2	This study
Streptomyces sahachiroi	Azinomycin B producer, wild type strain	ATCC
S. sahachiroi/Δ*aziA2*	*Streptomyces sahachiroi* with deletion of *aziA2*	This study
**Plasmids**		
pGEM®T-easy vector	*E. coli* general cloning vector, amp^r	Promega, USA
pKC1139	*Streptomyces*-*E. coli* bifunctional vector, Apr^r	Bierman *et. al*.,1992 [3]
pKC-AziA2	Disruption recombinant plasmid for *aziA2*	This study

Table S2: List of primers used in this study

Primers	Sequence (5'-3')*
**Gene disruption primers**	
aziA2UF	AAGCTTGACTACGAAGACTACATGCCCC
aziA2UR	TCTAGAGTCGGCGAGGTCCGGTGCGCCC
aziA2DF	TCT AGAGTCCCAGACCCTGCTGATGCAGG
aziA2DR	GAATTCAGGGACCGCGGCTCCCGCTCCGT
TsrF	TCTAGAGATCAAGGCGAATACTTCATATG
TsrR	TCTAGAACGAATCGAGGTCGAGGAACCGAG
**Primers for detection of genotype**	
aziA2 probe-F	TGATGCAGGAGCTGAGCACCGCCT
aziA2 Probe R	GACGATCTCCGCGAAGGGCAGCT

* Restriction sites are underlined

References

1. Kieser, T.; Bibb, M.; Butter, M.; Chater, K. F.; Hopwood, D. A., *Practical Streptomyces Genetics*. The John Innes Foundation: Norwich, UK, 2000.

2. Simon, R., Priefer U. and Pühler A., A Broad Host Range Mobilization System for In vivo Genetic Engineering: Transposon Mutagenesis in Gram Negative Bacteria. *Biotechnol.* **1983,** *1,* 784–791.

3. Bierman, M.; Logan, R.; O'Brien, K.; Seno, E. T.; Nagaraja Rao, R.; Schoner, B. E., Plasmid Cloning Vectors for the Conjugal Transfer of DNA from *Escherichia coli* to *Streptomyces spp*. *Gene* **1992,** *116,* 43–49.
